# The complete chloroplast genome of *Agave fourcroydes*

**DOI:** 10.1080/23802359.2021.1950065

**Published:** 2021-07-19

**Authors:** Xu Qin, Xinli Yang, Xing Huang, Xianya Huang, Xinyi Peng, Ming Liu, Tao Chen, Kexian Yi

**Affiliations:** a Guangxi Subtropical Crops Research Institute, Nanning, PR China; b College of Tropical Crops, Hainan University, Haikou, PR China; c Environment and Plant Protection Institute, Chinese Academy of Tropical Agricultural Sciences, Haikou, PR China

**Keywords:** *Agave fourcroydes*, chloroplast genome, phylogenetic tree

## Abstract

*Agave fourcroydes* (henequen) is the only cultivated *Agave* species in the Yucatan Peninsula, which is mainly used for fiber production. In the present study, we have successfully assembled the chloroplast (cp) genome of *A*. *fourcroydes*. The full length of the cp genome is 157,291 bp with a GC content at 37.8%. The cp genome is constructed with an inverted repeat region a (IRa) of 26,573 bp, a small single copy region (SSC) of 18,230 bp, an inverted repeat region b (IRb) of 26,573 bp and a large single copy region (LSC) of 85,915 bp. The annotation result reveals 132 genes on the cp genome, including 86 protein-coding genes, 38 tRNAs and 8 rRNAs. Phylogenetic tree reveals that *A*. *fourcroydes* is closely related with *A. sisalana*.

*Agave fourcroydes* Lemaire 1864 (henequen) is the only cultivated *Agave* species in the Yucatan Peninsula, which is mainly used for fiber production (Colunga-Garcíamarín et al. 1999; Piven et al. 2001). There are a large amount of leaf juice and fibrous waste after fiber extraction. It has been reported that the leaf juice of *A. fourcroydes* could be used as feedstock for ethanol production, which makes it of great potential in warming and drying regions around the world (Cáceres-Farfán et al. 2008; Yang et al. 2015). Till now, a series of studies have revealed the phylogenetic relationships of more than 37 *Agave* species (Huang et al. 2018; Jiménez-Barron et al. 2020). However, the phylogenetic relationship and systematic position of *A. fourcroydes* still remain ambiguous at the chloroplast (cp) genome level. Thus, we conducted the Illumina sequencing work for the assembly of its cp genome, with the purpose to reveal its systematic position and benefit future studies on *Agave* cp.

The young leaves of *A. fourcroydes* were cut from a three-year-old plant from the germplasm garden of Guangxi Subtropical Crops Research Institute, Nanning, China (22.90°N, 108.33°E). The specimen was deposited in Herbarium of Guangxi Subtropical Crops Research Institute (http://www.gxrzs.com/, Tao Chen, 15607718198@wo.cn) under the voucher number HGS-jm2020012. The leaves were ground in liquid nitrogen for the extraction of total genomic DNA by the modified CTAB method (Doyle and Doyle 1987). DNA sample was used for library construction and Illumina sequencing in Biozeron Biotech (Shanghai, China). The Illumina NovaSeq platform was selected for paired-end short reads sequencing, which generated a total of 5.28 Gb raw data. The raw data was submitted to SRA under the accession of PRJNA705498. The cp genome was assembled with the NOVOPlasty software and then gap filled with the GapCloser software (Luo et al. 2012; Dierckxsens et al. 2017). The cp genome was further annotated and corrected by DOGMA and Geneious v11.0.3, respectively (Wyman et al. 2004; Kearse et al. 2012). The full cp genome sequence of *A. fourcroydes* was submitted to GenBank under the accession of MW540496.

The full length of the assembled *A. fourcroydes* cp genome is 157,291 with a GC content at 37.84%. The cp genome contain four sequence regions, including IRa (26,573 bp), IRb (26,573 bp), SSC (18,230 bp) and LSC (85,915 bp). The annotation result reveals 132 genes on the cp genome, including 86 protein-coding genes, 38 tRNAs and 8 rRNAs.

We selected a total of 29 cp genome sequences for phylogenetic analysis. Among these, there are 26 species in Agavoideae and 3 other species (*Albuca kirkii*, *Nolina atopocarpa* and *Oziroe biflora*) as outgroup (McKain et al. 2016; Lee et al. 2019; Jin et al. 2020). These full sequences were aligned by the MAFFT software (Katoh and Standley 2013). After which, a maximum likelihood phylogenetic tree was constructed by MEGA7 software with bootstrap values of 1000 replicates (Kumar et al. 2016). The result revealed that *A. fourcroydes* is closely related with *A. sisalana* (Figure 1). This study would expand the number of plant chloroplast genomes and benefit future studies related to *Agave* chloroplast.

**Figure 1.**
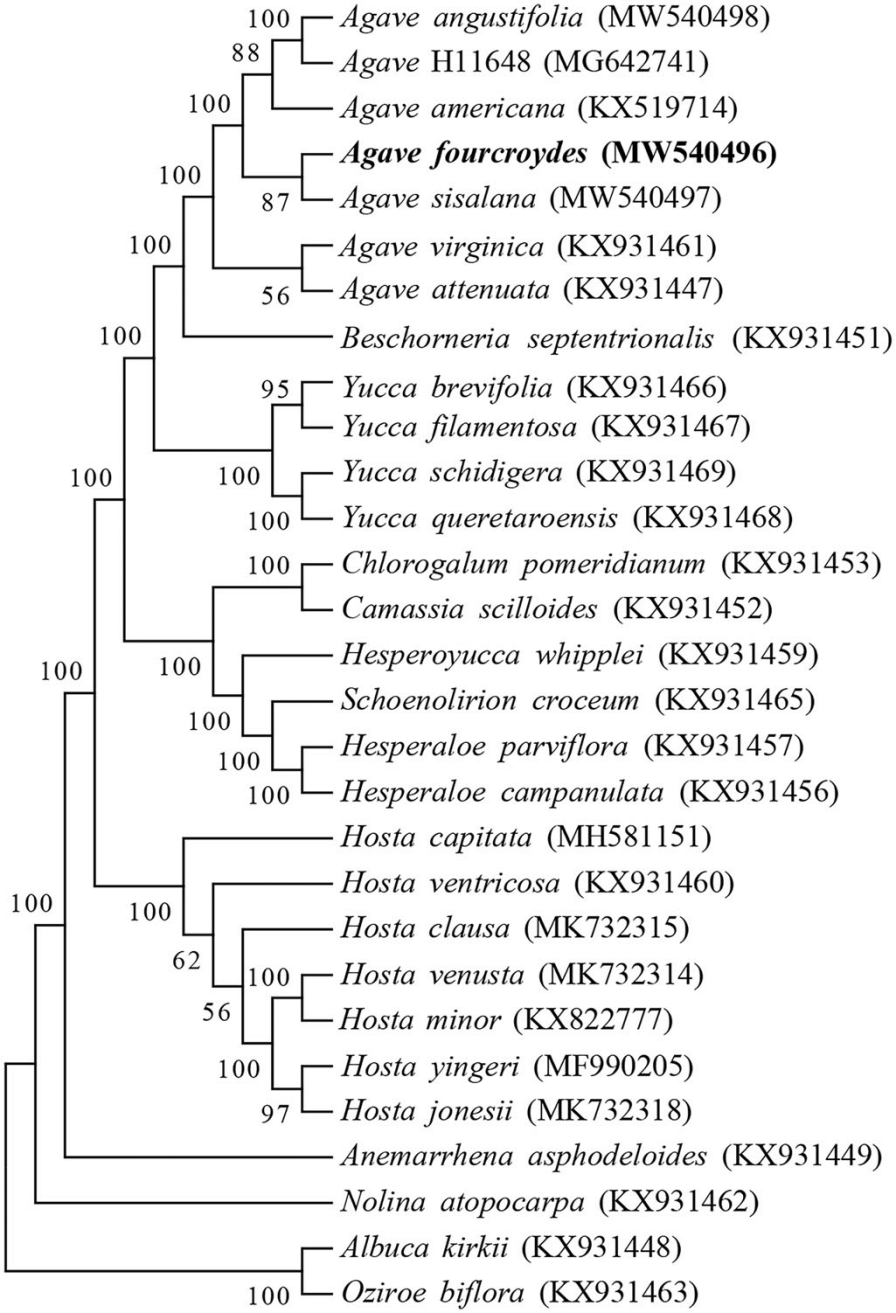
Phylogenetic tree of 29 chloroplast genomes.

## Data Availability

The genome sequence data that support the findings of this study are openly available in GenBank of NCBI at (https://www.ncbi.nlm.nih.gov/nuccore/) under the accession MW540496. The associated BioProject, SRA and Bio-Sample numbers are PRJNA705498, SRX10192643 and SAMN18090721, respectively.

## References

[CIT0001] Cáceres-Farfán M, Lappe P, Larqué-Saavedra A, Magdub-Méndez A, Barahona-Pérez L. 2008. Ethanol production from henequen (*Agave fourcroydes* Lem.) juice and molasses by a mixture of two yeasts. Bioresour Technol. 99(18):9036–9039. 18524573 10.1016/j.biortech.2008.04.063

[CIT0002] Colunga-Garcíamarín P, Coello-Coello J, Eguiarte LE, Piñero D. 1999. Isozymatic variation and phylogenetic relationships between henequen (Agave fourcroydes) and its wild ancestor *A. angustifolia* (Agavaceae). Am J Bot. 86(1):115–123. 21680351

[CIT0003] Dierckxsens N, Mardulyn P, Smits G. 2017. NOVOPlasty: de novo assembly of organelle genomes from whole genome data. Nucleic Acids Res. 45(4):e18. 28204566 10.1093/nar/gkw955 PMC5389512

[CIT0004] Doyle JJ, Doyle JL. 1987. A rapid DNA isolation procedure from small quantities of fresh leaf tissues. Phytochem Bull. 19:11–15.

[CIT0005] Huang X, Wang B, Xi J, Zhang Y, He C, Zheng J, Gao J, Chen H, Zhang S, Wu W, et al. 2018. Transcriptome comparison reveals distinct selection patterns in domesticated and wild Agave species, the important CAM plants. Int J Genom. 2018:5716518. 10.1155/2018/5716518 PMC6282153 30596084

[CIT0006] Jiménez-Barron O, García-Sandoval R, Magallón S, García-Mendoza A, Nieto-Sotelo J, Aguirre-Planter E, Eguiarte LE. 2020. Phylogeny, diversification rate, and divergence time of *Agave sensu* lato (Asparagaceae), a group of recent origin in the process of diversification. Front Plant Sci. 11:536135. 33240289 10.3389/fpls.2020.536135 PMC7680843

[CIT0007] Jin G, Huang X, Chen T, Qin X, Xi J, Yi K. 2020. The complete chloroplast genome of agave hybrid 11648. Mitochondrial DNA Part B. 5(3):2345–2346. 33457785 10.1080/23802359.2020.1775145 PMC7781945

[CIT0008] Katoh K, Standley DM. 2013. MAFFT multiple sequence alignment software version 7: improvements in performance and usability. Mol Biol Evol. 30(4):772–780. 23329690 10.1093/molbev/mst010 PMC3603318

[CIT0009] Kearse M, Moir R, Wilson A, Stones-Havas S, Cheung M, Sturrock S, Buxton S, Cooper A, Markowitz S, Duran C, et al. 2012. Geneious Basic: an integrated and extendable desktop software platform for the organization and analysis of sequence data. Bioinformatics. 28(12):1647–1649. 22543367 10.1093/bioinformatics/bts199 PMC3371832

[CIT0010] Kumar S, Stecher G, Tamura K. 2016. MEGA7: molecular evolutionary genetics analysis version 7.0 for bigger datasets. Mol Biol Evol. 33(7):1870–1874. 27004904 10.1093/molbev/msw054 PMC8210823

[CIT0011] Lee SR, Kim K, Lee BY, Lim CE. 2019. Complete chloroplast genomes of all six Hosta species occurring in Korea: molecular structures, comparative, and phylogenetic analyses. BMC Genomics. 20(1):833. 31706273 10.1186/s12864-019-6215-y PMC6842461

[CIT0012] Luo R, Liu B, Xie Y, Li Z, Huang W, Yuan J, He G, Chen Y, Pan Q, Liu Y, et al. 2012. Soapdenovo2: an empirically improved memory-efficient short-read de novo assembler. Gigascience. 1(1):18. 23587118 10.1186/2047-217X-1-18 PMC3626529

[CIT0013] McKain MR, McNeal JR, Kellar PR, Eguiarte LE, Pires JC, Leebens-Mack J. 2016. Timing of rapid diversification and convergent origins of active pollination within Agavoideae (Asparagaceae). Am J Bot. 103(10):1717–1729. 27793858 10.3732/ajb.1600198

[CIT0014] Piven NM, Barredo-Pool FA, Borges-Argáez IC, Herrera-Alamillo MA, Mayo-Mosqueda A, Herrera-Herrera JL, Robert ML. 2001. Reproductive biology of henequen (*Agave fourcroydes*) and its wild ancestor *Agave angustifolia* (Agavaceae). I. Gametophyte development. Am J Bot. 88(11):1966–1976. 21669630

[CIT0015] Wyman SK, Jansen RK, Boore JL. 2004. Automatic annotation of organellar genomes with dogma. Bioinformatics. 20(17):3252–3255. 15180927 10.1093/bioinformatics/bth352

[CIT0016] Yang X, Cushman JC, Borland AM, Edwards E, Wullschleger SD, Tuskan GA, Owen NA, Griffiths H, Smith JA, De Paoli HC, et al. 2015. A roadmap for research on crassulacean acid metabolism (CAM) to enhance sustainable food and bioenergy production in a hotter, drier world. New Phytol. 207(3):491–504. 26153373 10.1111/nph.13393

